# The Experiment Factory: Standardizing Behavioral Experiments

**DOI:** 10.3389/fpsyg.2016.00610

**Published:** 2016-04-26

**Authors:** Vanessa V. Sochat, Ian W. Eisenberg, A. Zeynep Enkavi, Jamie Li, Patrick G. Bissett, Russell A. Poldrack

**Affiliations:** ^1^Program in Biomedical Informatics, Stanford UniversityStanford, CA, USA; ^2^Department of Psychology, Stanford UniversityStanford, CA USA

**Keywords:** web-experiments, behavior, docker, assessment, reproducibility, experiments

## Abstract

The administration of behavioral and experimental paradigms for psychology research is hindered by lack of a coordinated effort to develop and deploy standardized paradigms. While several frameworks (Mason and Suri, 2011; McDonnell et al., 2012; de Leeuw, 2015; Lange et al., 2015) have provided infrastructure and methods for individual research groups to develop paradigms, missing is a coordinated effort to develop paradigms linked with a system to easily deploy them. This disorganization leads to redundancy in development, divergent implementations of conceptually identical tasks, disorganized and error-prone code lacking documentation, and difficulty in replication. The ongoing reproducibility crisis in psychology and neuroscience research (Baker, 2015; Open Science Collaboration, 2015) highlights the urgency of this challenge: reproducible research in behavioral psychology is conditional on deployment of equivalent experiments. A large, accessible repository of experiments for researchers to develop collaboratively is most efficiently accomplished through an open source framework. Here we present the Experiment Factory, an open source framework for the development and deployment of web-based experiments. The modular infrastructure includes experiments, virtual machines for local or cloud deployment, and an application to drive these components and provide developers with functions and tools for further extension. We release this infrastructure with a deployment (http://www.expfactory.org) that researchers are currently using to run a set of over 80 standardized web-based experiments on Amazon Mechanical Turk. By providing open source tools for both deployment and development, this novel infrastructure holds promise to bring reproducibility to the administration of experiments, and accelerate scientific progress by providing a shared community resource of psychological paradigms.

## 1. Introduction

Experimental paradigms are a common means by which we quantify human behavior. Given this central role, there would ideally be a coordinated effort to develop and deploy standardized paradigms. Unfortunately there is no openly available, extensible effort, and thus behavioral datasets tend to be small, and not directly comparable across studies. The technology available is an important limiting factor; while several frameworks have been developed for specific steps in the experimentation process (e.g., jsPsych de Leeuw, 2015 for experiment creation, Psiturk McDonnell et al., 2012 for deployment), these tools require expertise with programming or the command line, and lack an integrated framework. Additionally, there is currently no large, open repository of paradigms that can serve as a resource and standard for the field. Without such a resource, individual labs must either spend unnecessary time coding tasks, or pay for commercial products that provide a battery of psychological assessments. Behavioral science can benefit from a more concentrated and conscious effort to adopt modern technology, including instant online access to deploying surveys (e.g., www.surveymonkey.com, www.qualtrics.com), integration with social media (e.g., Facebook, Twitter), and a general movement toward a fast, broad collection of data (Mason and Suri, 2011).

With the explosion of experimental tools and behavioral paradigms to investigate mental function, there is a need for standardization and harmonization across the field. Historically, behavioral experimentation in psychology has relied upon a small number of libraries and software for the generation of behavioral tasks: E-Prime (Schneider et al., 2012), Psych Toolbox (Brainard, 1997; Matlab), psychopy (Peirce, 2007; Python), jsPsych (de Leeuw, 2015; JavaScript). Within this current system, sharing of code is infrequent, documentation tends to be sparse, and formal testing is almost nonexistent. The detriments to the research process are extensive. First, independent development of standard paradigms by multiple individuals leads to redundancy of effort and increases the probability of coding and conceptual errors in task design. Even when labs share their code there is the potential for propagation of error across labs if the benefactors do not rigorously vet the paradigms. Second, lack of an open repository of paradigms slows the establishment of standardized behavioral measures; while certain paradigms may be popular in subsections of psychology, they may be wholly unknown in others. By the same token, lack of open sharing slows the adoption and vetting of new paradigms, since their use either requires independent development or acquiring them from the original lab, both of which delay reproduction and extension of the original work. Finally, lack of standardization complicates interpretation of paradigms by potentially conflating theoretically meaningful differences in task design with accidents of implementation.

A promising trend to address these issues is the growth of browser-based experimentation, such that behavioral paradigms are delivered online or offline through a web browser. While some toolboxes are limited to running with specific software (Brainard, 1997; Schneider et al., 2012), delivery of experiments over the web using platforms like Amazon Mechanical Turk have become increasingly popular (Stewart et al., 2015), with results consistent with in-laboratory settings (Woods et al., 2015). For example, the Many Labs study (Klein et al., 2014) successfully replicated 10 out of 13 selected classical paradigms using 36 independent samples to show that effects are robust across samples and settings. The use of the web necessitates a move to standard web technologies including JavaScript, HTML, and CSS. The primary benefit of web-based experimentation is that experiments can be delivered across platforms (e.g., computers, mobile phones, tablets, fMRI projected screens), and environments (controlled and uncontrolled settings). Companies have noticed this trend, and there are a number of pay-for-service products available (e.g., http://www.millisecond.com offers “Implicit,” and http://www.nightingaleapp.com offers an app for data collection in organizations). While these solutions are ideal for the controlled collection, organization, and delivery of primarily survey-based data, these products are not ideal for researchers who generally desire the transparency and ultimate control over their experiments afforded by the use of open source software. Additionally, the scope of tasks that can be deployed is limited. The ideal would be to have published paradigms, surveys, and games that have precise response latency measurement, along with auditory and visual stimuli. The ideal would be for this public resource to be open, flexible, and under the control of the researchers that use it, which is not the case for commercial solutions. The development of an open-source equivalent, on the other hand, would meet these requirements.

A wide range of standard infrastructures are in development to help with this task. Just Another Tool for Online Studies (JATOS) provides infrastructure to set up a local or server-based set of JavaScript experiments with a corresponding database, but does not address the issue of standardizing or re-using paradigms (Lange et al., 2015). The Project Implicit Framework (PIP) (http://www.peoplescience.org) is a modular framework that also deploys JavaScript experiments, but requires significant expertise to develop and set up components of the application. Psiturk (McDonnell et al., 2012) is a Python-based web framework (based on the Flask micro-framework) that researchers can use to develop experiments and deploy on Amazon Mechanical Turk, but is limited to that implementation, and requires researchers to develop their own paradigms.

A commercial solution, E-Prime (Schneider et al., 2012) must be mentioned in that it has been well adopted into the community. While E-Prime offers equivalent fine-tuned control of stimuli presentation, the requirement of a USB license to run it, inability to serve experiments online (e.g., on Amazon Mechanical Turk), and substantial learning to create experiments (MacWhinney et al., 2001) make it ill-suited for widespread and easy deployment of experimental paradigms. MacWhinney et al. (2001) have the System for Teaching Experimental Psychology (STEP) that is intended to maximize the use of E-Prime for both teaching and experimental purposes. STEP includes documentation and E-Prime scripts for a variety of classic and commonly used paradigms. However, because it uses E-Prime, it inherits the problems of using E-Prime, so it is also ill-suited for widespread adoption and open collaboration.

Finally, Tatool is a web-based tool for researchers to create and run experiments, offering modern and accessible experiment generation and analysis. While Tatool is easy to use and a great contribution to open-source experiment technology, it does not provide standardization to the development of experiments, sharing and development of a common resource, or integration with existing deployment options (e.g., Psiturk; Makin, 2016). Here we present the Experiment Factory, an open source framework for the development and deployment of web-based experiments. Central to the Experiment Factory is a collection of over 80 standardized paradigms, which are immediately available for use, and can be easily extended and improved upon through open-source collaboration.

## 2. The experiment factory

An essential and often overlooked feature of research workflow is the distinction between developer and user. A developer is a researcher interested in creating experimental paradigms and infrastructure, while a user simply wants to use the paradigms. While some researchers aim to dig deep into the code for an application, others simply want to use it, and a successful infrastructure must serve both. Toward this aim, the Experiment Factory employs a modular strategy, providing separate components (Github repositories) for experiments, surveys, battery, and deployments (see Section 4, The Experiment Factory Infrastructure), and allows researchers to deploy experiments into current tools that researchers already find useful (e.g., Psiturk, see **Table 3** for Glossary of terms), and under different likely scenarios like not having access to an internet connection, or needing to save data to a private database. First we will outline some use case scenarios to describe the motivation behind this work, followed by an outline of the infrastructure in more detail. We have made available a “getting started” guide to better familiarize users of the framework (http://expfactory.readthedocs.org/en/latest/getting-started.html).

## 3. The experiment factory use cases

### 3.1. Deployment of experiments

The Experiment Factory aims to offer both flexibility and structure. A researcher has complete control over the deployment environment (local or cloud), along with the infrastructure used for the deployment (see Section 4, The Experiment Factory Infrastructure). Under any circumstance, the researcher has complete control over the set of experiments that are selected, and the resulting data are provided in several output formats. For this manuscript, we will walk through several use cases. When we refer to a module such as "experiments," we are referring to a Github repo, and more information is available about these components in Section 4.

#### 3.1.1. Local deployment of experiments

The most basic use case is when a researcher wants to run participants locally through the paradigms already included in the experiments module. This approach is easily accomplished using the expfactory command line tool (Section 4.1 The Experiment Factory Software). Using the tool, a researcher can, in one line of code, select experiments, define a participant unique ID, and bring up a web interface to deploy a set of connected experiments called a “battery” (e.g., “expfactory --run --experiments stroop,nback --subid UID001”). In the case that the user wants to save a static folder to run later, the argument “--generate” can be used in a similar fashion. While the default behavior is to use the latest experiments and battery templates from the repositories, a researcher can ensure that experiments and battery code remains constant by saving (cloning) the repositories to a local machine, and providing the paths to the folders as arguments to the tool (see the docs at expfactory.readthedocs.org for details). The data are downloaded to the local machine directly in the browser after the experiment completes, and named according to the participant unique ID.

#### 3.1.2. Deployment of experiments using psiturk

The Psiturk infrastructure is a well-developed platform for deploying experiments on Mechanical Turk. Due to its substantial user base and documentation, it was imperative that our experiments be easily deployable to Psiturk. Using the expfactory command line tool without any arguments, a user can open up a web interface to choose experiments, a database specification, and a deployment. After selection of these variables, either a folder or file to run a virtual machine is provided to the user. This functionality is also possible using the command line tool with “expfactory --generate --experiments stroop,nback --folder /home/output.”

#### 3.1.3. Local modification of experiment or battery

Researchers may want to use the experiments included in the experiments module as a “base paradigm,” but make modifications for their own purposes (e.g., change the stimuli images, increase the trial length). Similarly, researchers may want to supplement the paradigms available with their own experiments. If a set of experiments have already been downloaded as a set of folders, modifying a paradigm simply requires changing the files in a specific experiment folder. While this currently requires some JavaScript coding knowledge, we plan to allow modification of a restricted set of experiment variables through variables in the configuration file of an experiment (Table 1) in the future.

**Table 1:** **The fields required in the standard config.jsonž for an Experiment Factory Experiment**.

**Field name**	**Requirement level**	**Rationale**	**Example**
name	Not required, warning	Descriptive label of experiment	Antisaccade
run	Required, not valid without	Scripts required for the experiment to run	experiment.js style.css
exp_id	Required, not valid without	Unique identifier	Antisaccade
cognitive_atlas_concept_id	Not required, warning	Mapping of experiment to cognitive concepts it measures	trm_4b1968619b00b
contributors	Not required	Credit and source of help	Ian Eisenberg Vanessa Sochat Zeynep Enkavi
time	Required, not valid without	Run time in minutes	8
experiment_variables	Not required	Variables for allocation of credit or reward	reaction_time
reference	Not required, warning	Full documentation of paradigm	doi:10.1006/cogp.1999.0734
notes	Not required	Additional information to capture	Should not wear glasses
publish	Required, not valid without	Ready for deployment	True
template	Required, not valid without	Experiment library base	jspsych

The definition of files and a template are essential to validate the function of the experiments. The examples for each field above are limited and not in JSON, full config.json are available for inspection in the experiment folders (https://github.com/expfactory/expfactory-experiments).

#### 3.1.4. Development of experiments and infrastructure

The Experiment Factory uses an open source development strategy, meaning that all code is publicly available on Github (http://www.github.com/expfactory), and contributions are made to any of the code bases via forking of repositories and pull requests. Full documentation about this process is available (http://expfactory.readthedocs.org/en/latest/development.html). The code base has been developed and tested on Linux (Ubuntu) and Mac OS.

#### 3.1.5. Experiment development

Contributing a new experiment constitutes adding a new folder to the experiments repository that meets the minimal requirements for expfactory (i.e., including a JavaScript file to run the experiment, and a configuration file to specify meta-data). Additional script and style files, along with images, sound, or other files necessary for deployment, are optional. We provide an experiment template for researchers to start with, and a recommended strategy is to copy and make modifications to a similar experiment that already exists. Development of an experiment means some familiarity with JavaScript and HTML/CSS, and ability to collaboratively work on Github. We provide both best practices and detailed descriptions about the required variables for the configuration file (Table 1). To test experiments, the user has several options. The Experiment Factory software command line tool can be run from within any experiment folder to open up a web browser to test a single experiment manually with “expfactory --preview,” to test a single experiment with an experiment robot using “expfactory --test,” or to validate the configuration file with “expfactory --validate.” See full documentation at http://expfactory.readthedocs.org.

#### 3.1.6. Documentation and infrastructure development

Infrastructure and methods are useless without proper documentation. The Experiment Factory uses the sphinx documentation tool, served with the Experiment Factory Github repository, meaning that it is built automatically when the code base is updated. This documentation standard uses restructured text syntax (rst) (http://docutils.sourceforge.net/rst.html). A set of pages have been written to supplement the functions that the module provides, and we also provide documentation for how to contribute to documentation (http://expfactory.readthedocs.org/en/latest/development.html#contributing-to-this-documentation). The Experiment Factory code is licensed under the MIT open source license, which is a highly permissive license that places few limits upon reuse. This ensures that the code will be usable by the greatest number of researchers, in both academia and industry. We welcome and encourage contributions for any of the Experiment Factory components from the larger community.

Complete tutorials and further details are provided in our development documentation (http://expfactory.readthedocs.org/en/latest/development.html).

## 4. The experiment factory infrastructure

**Figure 1:**
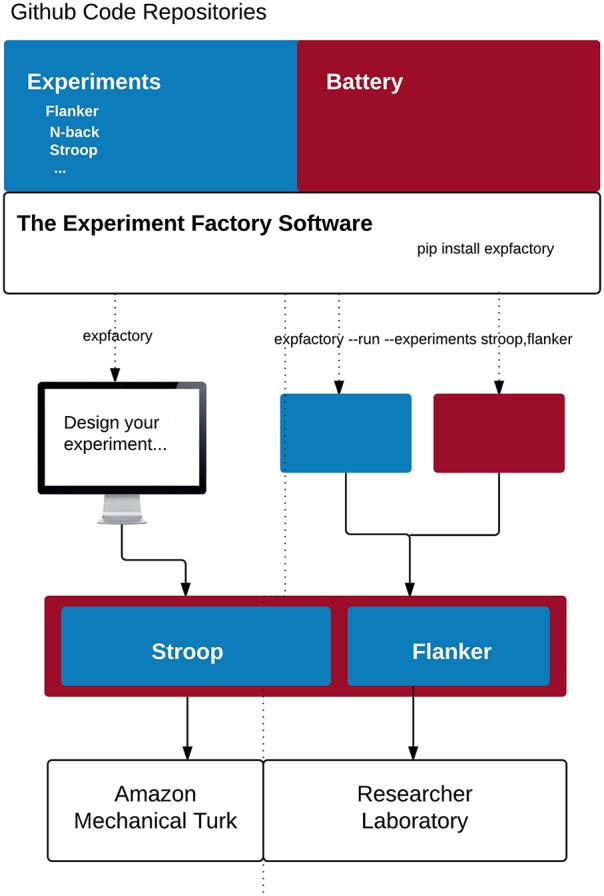
**The experiment factory core: Experiments, a battery skeleton, and the software are openly available on Github**. Installation of the Experiment Factory tool allows a researcher to run a sequence of experiments on the fly **(right)** or to generate a local folder or virtual machine to deploy experiments to Amazon Mechanical Turk using Psiturk **(left)**.

This section is intended for more technical readers and those interested in development of the Experiment Factory modules, and we provide a Glossary of terms (Table 3) to explain more technical jargon. The modular strategy of the Experiment Factory infrastructure means providing separate Github repositories for experiments, skeletons for a sequence of experiments (a “battery” of experiments), and deployments. The Experiment Factory aims to be agnostic when it comes to deployment, and to provide support for the current infrastructures that researchers find useful to deploy their experiments. For example, experiments can be easily deployed into a folder structure to plug in to Psiturk, either locally on a virtual machine, or served locally to study participants without an internet connection. These static components are separate from the software that drives integration of the components, and all components and software are completely open source (Github, RRID:SCR_002630), allowing for collaborative specialization. A researcher interested in developing an experiment need only add a new paradigm to the experiments repository, and it will be available to all applications that use the Experiment Factory. A researcher primarily interested in further developing the software can do so without needing to touch static components. Currently the entire system is available for deployment by any researcher, either locally or using a cloud server; ultimately, we also hope to provide a hosted version as a service open to all researchers. An overview of the infrastructure is included in Figure 1.

### 4.1. Experiment factory software

The controller of the Experiment Factory is the Experiment Factory Python software (https://github.com/expfactory/expfactory-python) that provides functions for working with components, testing and validating experiments, and generating the final battery output based on the users specifications. For example, after installing this tool, a researcher can, in one line, specify experiments and an optional subject unique id, and a browser opens with the rendered battery. Behind this simple functionality, the software is obtaining experiment and battery files from Github, validating the experiments, and parsing configuration files to render the users selected experiments into the correct HTML syntax. The finished HTML syntax, along with experiment code and static files, is saved in a temporary directory, and a web server is opened to run the experiments as a sequence.

The tool is also useful to developers in that any function in the software can be used in an external application. By way of being a Python Flask application (see **Table 3** for Term Glossary), running the executable also provides a RESTful API to serve experiment meta-data, which can be deployed in a local or cloud server environment. The application is easily installed with a package manager (https://pypi.python.org/pypi/expfactory), and developers can collaborate on this software to develop additional functions for use with the entire family of Experiment Factory components.

### 4.2. Experiment factory experiments and surveys

The Experiment Factory Experiments (https://github.com/expfactory/expfactory-experiments) and Surveys (https://github.com/expfactory/expfactory-surveys) are the core of the infrastructure: at the time of this publication there are more than 80 coded experiments and surveys available for deployment. Each experiment is a single folder in the expfactory-experiments Github repository that contains a data structure (config.json) file with a standard set of keyvalue relationships that provide meta-data on the experiment, and allow for its deployment. Table 1 provides an overview of fields, requirements, and examples, each of which is checked before an experiment is considered valid. For example, the definition of files necessary to run the experiment is essential for the expfactory-python tool to validate and deploy the experiments, and the definition of variables makes them available to the higher level applications. Each experiment is given a unique identifier, the “exp_id” variable, that coincides with the folder name in the Github repository. The boolean field “publish” makes it possible to quickly disable deployment of a particular experiment, and the fields “reference,” and “contributors” are important to allocate credit to developers. Finally, fields related to the Cognitive Atlas (Poldrack et al., 2011) allow for a common place to document details and references for the experiment, and define an experimental paradigm in an ontology that makes assertions about the cognitive concepts measured by the task. This means that a comparison can be made between tasks with regard to the processes or phenomena that are measured (e.g., finding all tasks that are thought to measure the construct of “response inhibition”). The “template” field specifies the library (e.g., JavaScript functions) that the experiment is coded in, such that the deployment template will be customized for the library. Although the initial release includes experiments coded using jsPsych, a Javascript library that simplifies experiment creation, the modular framework and specification of this template means that the infrastructure is ready to be extended to any web-based technology. This is extremely important to allow for development of experiments using the most up-to-date web-based technologies. Similar to experiments, each survey is a single folder in the expfactory-surveys Github repository. Surveys are fully specified by two files: a data structure (config.json) file identical to the one used for experiments and a TSV (survey.tsv) file which specifies the questions, responses and scoring of the survey.

### 4.3. Experiment factory battery

The Experiment Factory Battery (https://github.com/expfactory/expfactory-battery) is a simple skeleton into which Experiment Factory experiments can be deployed as a cohesive set of experiments, called a “battery.” The battery comes with a set of standard JavaScript and stylesheets common across the templates (e.g., jsPsych), meaning that code that is consistently re-used across paradigms can be added to this repository. The design of the battery allows immediate deployment to multiple infrastructures, including Psiturk (locally or via a virtual machine), to a local machine to run on the fly, or a Django (RRID:SCR_012855) application that can be served locally or on a server (expfactory-docker). This Django application drives the (www.expfactory.org) interface.

### 4.4. Experiment factory docker

**Figure 2:**
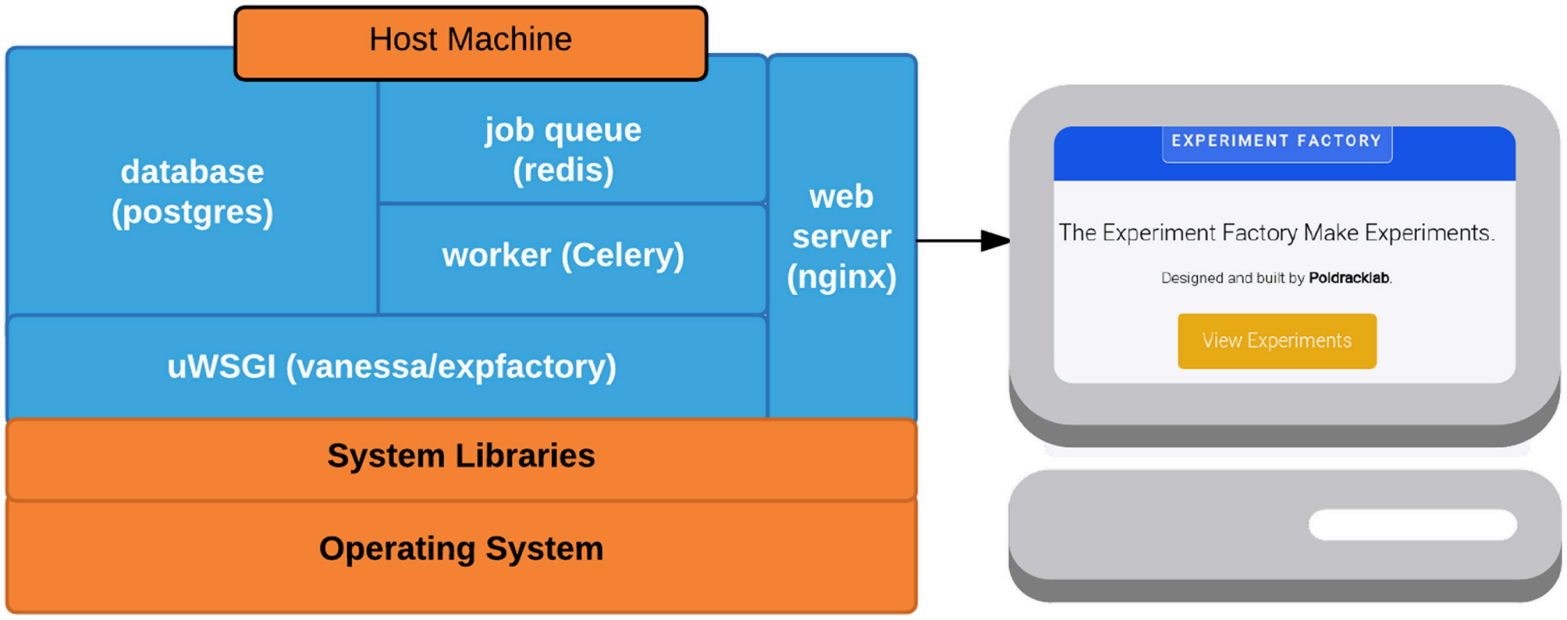
**Docker containers: Expfactory-docker includes the main application container (expfactory), a database for storing application data (postgres), a job queue (redis) and worker (Celery) for running computationally intensive tasks, and a web server (nginx) to serve the application to the web**.

One of the main goals of the Experiment Factory is to provide an ability to deploy experiments and collect data without any knowledge of programming, databases, or a command line. Under this requirement, download of a command line application is one step too many, and for this reason we developed a container-based application running at expfactory.org. The Experiment Factory Docker is a set of containers that serve a Django application that can be run locally or on a server to provide a login interface for labs to run experiments locally, or from the cloud. The application supports both http and https (secure connections). The application is also configured to deploy experiments to Amazon Mechanical Turk. The ease of deployment is thanks to Docker, an emerging container-based architecture that allows for development and deployment of applications in Linux containers (http://www.docker.com). Docker Compose (http://docs.docker.com/compose) is a controller for running multi-container applications such as the Experiment Factory, which uses separate containers for a nginx web server (nginx-proxy), a postgresql database (postgres), a Celery job manager worker to run time-intensive jobs (worker), a database for the jobs (redis), and the core application (expfactory), and protocol (uwsgi) for serving the application. An overview of these containers, along with the images on the Docker Hub, are provided in Table 2 and Figure 2, and a summary of terms are provided in a glossary (Table 3). For a more secure deployment (e.g., expfactory.org), it is recommended to link the application to a separate database with an encrypted connection over running the postgres container on the same server. Django (https://www.djangoproject.com/) is a Python-based framework that comes with a strong user base, well-developed plugins for authentication, security, and a backend database, and if desired, the Django application could be run independently from Docker.

**Table 2:** **Docker Containers utilized in expfactory-docker to run the www.expfactory.org**.

**Container name**	**Purpose**	**Image**
expfactorydocker_uwsgi_1	Django application, and uwsgi protocol for serving it	expfactory
expfactorydocker_db_1	Postgresql database for Django application and storing results	postgres
expfactorydocker_nginx_1	“Engine X” web server	nginx
expfactorydocker_worker_1	Celery worker for running tasks	expfactory
expfactory_redis_1	Redis database for tasks, serialized as JSON	redis

The container images are downloaded from the Docker hub. The container image “expfactory” corresponds to the expfactory-docker repository, and is built automatically from this source. For a more substantial deployment, the database can be external to the instance (e.g., Amazon RDS) with an encrypted connection.

**Table 3:** **Glossary of terms for technical jargon, software, and tool references**.

**Term**	**Definition**
Amazon Mechanical Turk (MTurk)	A platform provided by Amazon Web Services to allow individuals (Requesters) to deploy “human intelligence tasks,” or computer-based tasks that are difficult for computers, for other people to complete
Battery	A set of experimental paradigms presented in sequence to a study participant
Celery	A distributed task queue to allow for scheduling of function executions on a server
Continuous Integration	The continuous testing of functions in code whenever a change is made to ensure functionality does not break with changes
Cognitive Atlas	A collaborative knowledge base of ideas (e.g., cognitive concepts and experimental paradigms) in cognitive science
Docker	A container-based infrastructure to package an entire software environment (code, system libraries, files) for consistent deployment on different computers
Docker Compose	A tool for specification of how different Docker containers work together to build an application with multiple containers
Django	A Python-based web framework that makes it easy to extend Python-based functions into the web browser
Flask	A Python-based micro-framework with less stringent requirements than Django to extend Python-based functions into a web browser
jshint	A code analysis tool to check static (not running) JavaScript code for common errors
jsPsych	A JavaScript library for creating and running behavioral experiments
Psiturk	A Flask application to deploy web-based experiments to Amazon Mechanical Turk
Selenium	A tool to allow for programmatic control of web browsers
Sphinx	A documentation generation language for Python
Redis	An open source data structure store that can easily handle storage of different data structures for use with other applications
uWSGI	A tool to easily deploy web applications, including load balancing, process and task management, and monitoring

### 4.5. Experiment factory VM

Deployment of a battery to a virtual machine, whether locally or to the cloud, is made possible by the expfactory-vm repository. This repository contains Vagrantfiles that can be used with the Vagrant software (http://www.vagrantup.com) to run a local Virtual Machine, or one deployed via Amazon Web Services. The files can be used “as is” to deploy a battery with all experiments, or generated through the expfactory-python executable to allow a user to define a custom set of experiments.

## 5. Design and implementation choices

### 5.1. Modular framework for open science

The choice to use Github, and to separate the Experiment Factory into its underlying components (experiments, battery, docker, documentation, and vm) was a specific choice to allow specialization and collaboration in development. Github offers version control, management of code, and collaboration between teams, along with features such as reporting issues, discussing changes, and managing documentation. All versions of code are archived, and multiple features can be worked on simultaneously by any researcher with an internet connection. Github also provides Continuous Integration, or automatic testing of code, both for the experiments and expfactory-python, discussed next.

### 5.2. Software testing for the experiment factory

An essential component of software development is continuous testing of all functions whenever changes are made to the software in the case that a change breaks an essential functionality. This task, called Continuous Integration, can be done automatically when new changes are proposed to code on Github with services like CircleCI (https://circleci.com/) and Travis (https://travis-ci.com/). The base software to run the Experiment Factory (expfactory-python) is consistently tested in this fashion, however a significant challenge in the development of this infrastructure was ensuring functionality of the experiments themselves. An error in an experiment at run-time would end a battery, and must be avoided at all costs. Toward this goal, the Experiment Factory has several strategies for testing experiment code in a Continuous Integration environment. First, testing of experiments includes using jshint, a JavaScript quality tool, to parse experiment code files for static errors. The validation of experiments config.json data structures also occurs in the Continuous Integration environment, as does testing the experiments at run-time. This is made possible by using an automated web browser, selenium (http://www.seleniumhq.org), controlled by python functions from expfactory-python that respond to the stimuli, akin to running an experiment robot. When experiments are modified, the experiment robot is run over these changed experiments to ensure no run-time errors, triggering an error to fail the Continuous Integration tests if any errors are found. During this process, developers can discuss changes and issues using the standard forums for reporting issues and discussing development that are provided by Github. This collaborative coding environment has been an essential component for our group to develop, pilot, and discuss the application. Using version control was an essential factor for the Experiment Factory to follow the vision of reproducible science.

### 5.3. The cognitive atlas

The Cognitive Atlas (Poldrack et al., 2011) is an ontology that represents current knowledge about cognitive science. Integration of standard terms to describe the tasks, and consequently, the cognitive concepts that are measured by them, allows for researchers to map all experiments into a common space, and use a common language to describe the behavior and phenomena that are being measured. This means that, for example, a researcher can quickly find experiments that are asserted to measure “risk seeking,” and such a feature is not only important for definition of these experiments, but also for meta-analysis and reproducible science. The expfactory.github.io experiment portal, along with documentation and testing of experiments, offers a view to browse experiments based on the cognitive concepts that are measured, as defined in the Cognitive Atlas. Mapping experiments to the Cognitive Atlas and making assertions about the cognitive concepts measured by the experiments is powerful in that it allows researchers to select paradigms based on the specific cognitive functions that they are thought to measure.

## 6. Discussion

We have developed the Experiment Factory with a vision of open, collaborative science. The modular application is flexible to be used by both developers and researchers without development experience, and structured so that experiments must follow guidelines that will make them extendable to multiple frameworks. We have integrated the Cognitive Atlas as a way to provide structure in making assertions about what the experiments measure — any experiment tagged with a unique identifier in the Cognitive Atlas can immediately be compared to other experiments on the level of the cognitive concepts. While we are optimistic about our approach, there are several limitations.

### 6.1. Limitations

#### 6.1.1. Software versioning

A key challenge with any kind of deployment of this nature is software versioning. For example, the current experiments and battery are up to date with the most recent version of the JsPsych library, and upgrading this software would require developers to update current experiments. Thus, a standard in software development is to instill that care is taken to make available different versions of the software to support legacy implementations. Significant new releases of dependencies can be integrated when the developer community decides they are needed, and these same developers take responsibility for ensuring proper function of components. This is the rationale for Continuous Integration to run tests of the function of software, which has been implemented and provided by way of CircleCI (www.circleci.com) integration with Github.

#### 6.1.2. Ontology development

The Cognitive Atlas may not contain every experimental paradigm that would be desired, and so it might be required for a researcher to add a new experiment, extend documentation on an already defined experiment, or better develop the assertions about the cognitive concepts that the task measures. Ontology development is an ongoing process.

#### 6.1.3. Operating systems and browsers supported

We have tested the experiments fully on Chrome and Firefox browsers running on Linux and Mac OS systems. While we plan to develop a desktop application that will have cross platform support (i.e., including Windows), this desktop application is not yet available. In the meantime we encourage users to use the tools on Linux and Mac OS, in Chrome or Firefox, and to use a virtual machine for support on Windows systems.

#### 6.1.4. Community contributions

A significant challenge with the release of any new technology is adoption by the community. While we cannot ensure that researchers will be motivated to contribute new experiments, we are optimistic that The Experiment Factory will be well utilized. We have built the Experiment Factory from the ground up for ease and accessibility for both users and developers. Additionally, several groups have started using our software, contributing experiments, or expressing interest prior to any efforts to publicize the work.

A second point of concern is the quality of the implementations. We have developed the initial experiment set based on careful reading of published paradigms in the literature, along with significant feedback both from other groups of researchers and pilot studies. While a complete review of proper experimental design (e.g., MacWhinney et al., 2001) is outside of the scope of this technical paper, we have provided equivalent “best practices” for the development of a new paradigm to our documentation, and are optimistic that having an open source framework will ensure many eyes pass over the experiments, minimizing errors in implementations.

#### 6.1.5. Future development

The goal of the team of developers behind the Experiment Factory is to keep the set of tools and experiments modern. We believe that the same technology available and used in industry should be extended to researchers, and for this reason have chosen our current approach that uses modern technologies such as Docker, Amazon Mechanical Turk, and Amazon Web Services. Coinciding with this goal, we see a potential opportunity to deploy experiments via social networks such as Facebook, and have plans to develop this ability. We also see great potential in the development of experiments beyond the jsPsych framework, and have plans to do this. Currently we are developing a “games” extension to develop and deploy fun, interactive paradigms. Given the open nature of this work, we encourage and invite all researchers to join in the development of experiments, battery template, and deployment infrastructures.

## 7. Conclusion

The Experiment Factory is a modular infrastructure that applies a collaborative, open source framework to the development and deployment of psychology experiments. We are pleased to offer this as a resource for the larger community, and excited to further the development toward the needs of our users toward a vision of reproducible science.

## Author contributions

The Experiment Factory was conceived by authors VS and IE. The infrastructure idea was originated by VS with later contributions from IE. Experiments were developed by authors IE, AE, JL and tested by authors PB, IE, JL, and AE with contributions to the design from author VS. The Experiment Factory Battery skeleton was generated by authors IE and VS with minor extensions by AE. The Experiment Factory code base (python,vm,docker,surveys) was implemented by author VS. Manuscript textual content was prepared by authors VS, IE, and RP, with substantial feedback from PB and AE. Figures and manuscript formatting were produced by author VS. All authors approved of the final manuscript and are accountable for all aspects of the work.

## Funding

This work was supported by the National Institutes of Health (NIH) Science of Behavior Change Common Fund Program through an award administered by the National Institute for Drug Abuse (UH2DA041713). VS is supported by a William R. Hewlett Stanford Graduate Fellowship and a National Science Foundation Fellowship. PB was supported by the National Institute of Drug Abuse of the National Institutes of Health under award number F32 DA041773.

### Conflict of interest statement

The authors declare that the research was conducted in the absence of any commercial or financial relationships that could be construed as a potential conflict of interest.
